# COVID-19 Death Determination Methods, Minnesota, USA, 2020–2022^1^

**DOI:** 10.3201/eid3007.231522

**Published:** 2024-07

**Authors:** Lydia J. Fess, Ashley Fell, Siobhan O’Toole, Paige D’Heilly, Stacy Holzbauer, Leslie Kollmann, Amanda Markelz, Keeley Morris, Abbey Ruhland, Scott Seys, Elizabeth Schiffman, Haley Wienkes, Zachary Zirnhelt, Stephanie Meyer, Kathryn Como-Sabetti

**Affiliations:** Minnesota Department of Health, Saint Paul, Minnesota, USA (L.J. Fess, A. Fell, S. O’Toole, P. D’Heilly, S. Holzbauer, L. Kollmann, A. Markelz, K. Morris, A. Ruhland, S. Seys, E. Schiffman, H. Wienkes, Z. Zirnhelt, S. Meyer, K. Como-Sabetti);; Centers for Disease Control and Prevention, Atlanta, Georgia, USA (S. Holzbauer)

**Keywords:** COVID-19, 2019 novel coronavirus disease, coronavirus disease, severe acute respiratory syndrome coronavirus 2, SARS-CoV-2, viruses, respiratory infections, zoonoses, death certificate, vital statistics, mortality, epidemiology, Minnesota, United States

## Abstract

Accurate and timely mortality surveillance is crucial for elucidating risk factors, particularly for emerging diseases. We compared use of COVID-19 keywords on death certificates alone to identify COVID-19 deaths in Minnesota, USA, during 2020–2022, with use of a standardized mortality definition incorporating additional clinical data. For analyses, we used likelihood ratio χ^2^ and median 1-way tests. Death certificates alone identified 96% of COVID-19 deaths confirmed by the standardized definition and an additional 3% of deaths that had been classified as non–COVID-19 deaths by the standardized definition. Agreement between methods was >90% for most groups except children, although agreement among adults varied by demographics and location at death. Overall median time from death to filing of death certificate was 3 days; decedent characteristics and whether autopsy was performed varied. Death certificates are an efficient and timely source of COVID-19 mortality data when paired with SARS-CoV-2 testing data**.**

As of November 1, 2023, ≈1 million COVID-19 deaths have been reported in the United States (*1*) including >15,000 among Minnesota residents (*2*). The literature suggests that for the general population, COVID-19 death counts are probably underreported compared with excess mortality estimates (*3*–*8*) but may overrepresent White non-Hispanic and elderly populations (*9*). Extensive case-based mortality investigation is time and resource intensive, raising the question of how to balance accuracy, representativeness, and efficiency in a COVID-19 mortality surveillance system.

Accurate and timely mortality surveillance is a crucial tool for elucidating risk factors for death and also provides information for public health response, including policies associated with risk mitigation and high-risk populations (e.g., long-term care facility residents). COVID-19 mortality estimation methods have strengths and weaknesses (*10*). Death certificates are limited by the accuracy and consistency of completion by medical certifiers and by the evolving knowledge of SARS-CoV-2 pathogeneses and contribution to death. Similarly, excess deaths are limited by uncertainty around baseline and reported data. Early in the pandemic, rapid dissemination of mortality counts from healthcare systems and community surveillance were crucial to the public health response (*11*). However, officially filed death certificates, although often slower, provide valuable data about disease severity and disparity of mortality burden, as well as data for response planning (*10*). When available, death certificates are a valuable source of data for mortality surveillance for COVID-19 and other diseases (e.g., influenza) (*12*) and can also be relatively timely with access to provisional (not yet finalized) death certificates. Although previous work has analyzed codes from the International Classification of Diseases, Tenth Revision (ICD-10) listed on death certificates to determine appropriateness of COVID-19 inclusion, literature comparing death certificates with other forms of COVID-19 surveillance is lacking (*13*).

National organizations such as the Council of State and Territorial Epidemiologists (CSTE) provide guidance for COVID-19–associated mortality designation (*14*); however, mortality surveillance methods are determined primarily by individual jurisdictions and may differ. Early in the COVID-19 pandemic, Minnesota established a case investigation and death certificate–based mortality definition to enable systematic classification before the release of the first CSTE case definition.

To provide information for revisions to the state COVID-19–associated mortality definition, we evaluated the Minnesota Department of Health (MDH) COVID-19 mortality surveillance system. At the time of the analysis, Minnesota surveillance was more in-depth than the CSTE case definition and more robust than review of vital records alone, although it was more resource intensive. We sought to determine the effect of a more robust and resource-intensive case definition compared with the less resource-intensive case definition. To do so, we assessed the Minnesota surveillance system by calculating rates of agreement between COVID-19 deaths confirmed by the Minnesota standardized case definition and inclusion of COVID-19 on death certificates, as well as timeliness of death processing and reporting, throughout several years and phases of the pandemic. Our analysis was a surveillance evaluation of previously collected public health data obtained in accordance with Minnesota reportable disease statutes and is not subject to human subjects research review boards (National Archives and Records Administration, Title 45, Public Welfare, Code of Federal Regulations [annual edition] Sect. 46.102, Oct 1, 2020; and Minnesota Rules, 2018, Chapter 4605). 

## Methods

### Study Population

Our study population included decedents with confirmed COVID-19 deaths, as defined by the Minnesota definition of COVID-19 mortality, and deaths that were determined to not meet the COVID-19 death definition but included COVID-19 keywords on the death certificate (non–COVID-19 deaths). We excluded confirmed COVID-19 deaths without an available death certificate (e.g., Minnesota residents who died out of state). All decedents were Minnesota residents who died March 19, 2020 (first COVID-19 death in Minnesota), through December 31, 2022.

### COVID-19 Death Classification

Possible COVID-19 deaths were reported to MDH via provisional death certificates, by the MDH Unexplained Critical Illnesses and Deaths/Medical Examiner Infectious Deaths Surveillance (UNEX/MED-X) program (*15*,*16*), case interviews, laboratory results, and other sources including reports from providers, hospitals, long-term care facilities, and medical examiners. Death reports were linked with SARS-CoV-2 laboratory results reported to the Minnesota Electronic Disease Surveillance System and assessed by using the Minnesota COVID-19 mortality definition. Death certificates containing COVID-19 keywords (e.g., “COVID,” “SARS,” “coronavirus”) were pulled daily from the Minnesota Registration and Certification database and reviewed. Keywords were used in place of ICD-10 codes to enable identification before the ICD coding process and to avoid potential coding errors.

A death met the Minnesota definition of a COVID-19 mortality if the decedent had a positive laboratory SARS-CoV-2 RNA PCR or antigen test result before or after death and >1 of the following: COVID-19 was listed in either part I or part II of the cause of death section of the death certificate, or clinical history or autopsy findings were consistent with COVID-19 in the absence of an alternative cause of death as evaluated by MDH staff during the mortality investigation process (Appendix). For our analysis, a confirmed COVID-19 death was a death that met the Minnesota definition of COVID-19 mortality.

We further investigated deaths that occurred within 30 days of a COVID-19 infection but did not indicate COVID-19 on the death certificate, deaths that included conditional language on the death certificate such as history of COVID, deaths that included COVID-19 on the death certificate but the positive SARS-CoV-2 specimen collection date was >1 year before death, and deaths that included COVID-19 on the death certificate but had a potential alternative cause of death. When necessary, we consulted additional information (e.g., medical records and autopsy reports) to determine if a death met the case definition. Data for confirmed COVID-19 deaths and all other death certificates with COVID-19 keywords were managed in a REDCap (Research Electronic Data Capture) database (*17*,*18*).

For analyses, we created the following groups: confirmed COVID-19 deaths with COVID-19 keywords listed on the death certificate (confirmed COVID-19 deaths with death certificate), confirmed COVID-19 deaths that were determined to meet the Minnesota definition of mortality but did not include COVID-19 on the death certificate (confirmed COVID-19 deaths without death certificate), and deaths that included COVID-19 on the death certificate but did not meet the Minnesota definition of COVID-19 mortality (ruled-out COVID-19 deaths). We considered confirmed COVID-19 deaths with death certificate to have agreement between the Minnesota definition of COVID-19 mortality and the death certificate, and we considered confirmed COVID-19 deaths without death certificate and ruled-out deaths to have disagreement. We assessed timeliness of COVID-19 death investigations by calculating median number of days from date of death (DOD) to date of death certificate medical filing.

### Analyses

To assess death certificate agreement with the Minnesota definition of COVID-19 mortality, we compared all confirmed COVID-19 deaths with death certificates with confirmed COVID-19 deaths without death certificates and ruled-out COVID-19 deaths. We reviewed ruled-out deaths for death certificate language before analysis. Death certificates that clearly indicated that COVID-19 did not contribute to death (e.g., “viral infection, not COVID” listed in part I or II of the death certificate) were excluded from analysis. We approximated death certificate accuracy as agreement between inclusion of COVID-19 on the death certificate as a primary or contributing cause of death and the Minnesota definition of COVID-19 mortality. To calculate rates of agreement, we generated a 2 × 2 table of COVID-19 mortality definition and inclusion of COVID-19 on the death certificate.

We analyzed all measures by sex, age group, race/ethnicity, Minnesota region of residence (Minneapolis/Saint Paul metropolitan area or greater Minnesota), living setting, location of death, variant era, hospitalization history, underlying health conditions, autopsy status (whether performed), and median days between symptom onset (or if unavailable, date of specimen with positive SARS-CoV-2 result) and death. We defined variant eras by the week(s) at which the COVID-19 variant or lineage accounted for >50% of sequenced samples in Minnesota. To analyze categorial variables, we used likelihood χ^2^ or Fisher exact tests; for continuous variables, we used a median 1-way analysis. We conducted all analyses in SAS 9.4 (https://www.sas.com) and defined significance as p<0.05.

## Results

Our analysis included 14,004 deaths among Minnesota residents: 13,591 confirmed COVID-19 deaths (13,108 confirmed COVID-19 deaths with death certificate and 483 confirmed COVID-19 deaths without death certificate) and 413 ruled-out COVID-19 deaths. We excluded 59 ruled-out deaths because of language that indicated that COVID-19 did not contribute to death. Confirmed COVID-19 deaths most often occurred in persons who were male (54%) and >80 years of age (53%) (Table 1). Most confirmed COVID-19 decedents were White non-Hispanic (87%), were from the Minneapolis/Saint Paul metropolitan area (52%), and lived in a private residence (51%). About 64% persons with confirmed death had a known COVID-19–associated hospitalization before death, and death occurred during hospitalization for 50%. Only 3% of confirmed COVID-19 decedents underwent an autopsy; 95% had >1 documented underlying condition. Many (49%) of the confirmed COVID-19 deaths occurred before Alpha variant predominance in Minnesota. Median time between symptom onset and death was 14.0 days (interquartile range [IQR] 8.0–25.0 days).

**Table 1:** Patient demographic and disease history characteristics for COVID-19 deaths determined by using the Minnesota Department of Health case definition of COVID-19 mortality, March 19, 2020–December 31, 2022*

**Patient characteristic**	Total	Confirmed COVID-19 deaths	Ruled-out COVID-19 deaths
**Sex**			
** M**	7,518 (53.7)	7,336 (54.0)	182 (44.1)
** F**	6,486 (46.3)	6,255 (46.0)	231 (55.9)
**Age, y**			
**0–17**	19 (0.14)	14 (0.10)	5 (1.2)
**18–49**	561 (4.0)	529 (3.9)	32 (7.8)
**50–59**	920 (6.6)	903 (6.6)	17 (4.1)
**60–69**	1,921 (13.7)	1,883 (13.9)	38 (9.2)
**70–79**	3,176 (22.7)	3,098 (22.8)	78 (18.9)
**>80**	7,407 (52.9)	7,164 (52.7)	243 (58.8)
**Race/ethnicity**			
**American Indian/Alaska Native**	241 (1.7)	232 (1.7)	9 (2.2)
**Asian/Pacific Islander**	517 (3.7)	512 (3.8)	5 (1.2)
**Black/African American**	686 (4.9)	668 (4.9)	18 (4.4)
**Hispanic**	353 (2.5)	341 (2.5)	12 (2.9)
**Multiracial**	62 (0.44)	60 (0.4)	2 (0.48)
**Other or unknown**	22 (0.16)	21 (0.2)	1 (0.24)
**White non-Hispanic**	12,123 (86.6)	11,757 (86.5)	366 (88.6)
**Minnesota region**			
**Greater Minnesota**	6,665 (47.6)	6,501 (47.8)	164 (39.7)
**Minneapolis and Saint Paul metropolitan area**	7,339 (52.4)	7,090 (52.2)	249 (60.3)
**Living setting**			
**Private residence**	7,018 (50.1)	6,880 (50.6)	138 (33.4)
**Long-term care**	6,916 (49.4)	6,645 (48.9)	271 (65.6)
**Other†**	70 (0.50)	66 (0.5)	4 (0.97)
**Location of death**			
**Hospital inpatient**	6,827 (48.8)	6,748 (49.7)	79 (19.1)
**Congregate living**	5,497 (39.3)	5,245 (38.6)	252 (61.0)
**Other‡**	1,680 (12.0)	1,598 (11.8)	82 (19.9)
**Hospitalization history**			
**Hospitalized**	8,860 (63.3)	8,657 (63.7)	203 (49.2)
**No/unknown**	5,144 (36.7)	4,934 (36.3)	210 (50.9)
**Autopsy status**			
**Yes**	343 (2.5)	343 (2.5)	0
**No/unknown**	13,661 (97.6)	13,248 (97.5)	413 (100.0)
**Variant era**			
**Pre-Alpha**	6,853 (48.9)	6,697 (49.3)	156 (37.8)
**Alpha**	857 (6.1)	778 (5.7)	79 (19.1)
**Delta**	2,851 (20.4)	2,799 (20.6)	52 (12.6)
**Omicron BA.1**	1,785 (12.8)	1,754 (12.9)	31 (7.5)
**Omicron BA.2**	335 (2.4)	314 (2.3)	21 (5.1)
**Omicron BA.4/BA.5**	1,323 (9.5)	1,249 (9.2)	74 (17.9)
**Underlying conditions status**			
**Yes**	13,300 (95.0)	12,921 (95.1)	379 (91.8)
**No**	192 (1.4)	188 (1.4)	4 (1.0)
**Unknown**	512 (3.7)	482 (3.6)	30 (7.3)
**Median onset date to death (days, IQR)**	15.0 (8.0–27.0)	14.0 (8.0–25.0)	82.0 (30.0–185.0)
**Total**	14,004	13,591 (97.1)	413 (3.0)

*Values are no. (%) except as indicated. IQR, interquartile range.†Other includes sheltered and unsheltered homeless, jail/prison, dormitories, and other settings. ‡Other includes decedents who died at home, in the emergency department, or in other settings, such as at another private residence.

### Timeliness of COVID-19 Death Processing

The median number of days between DOD and date of death certificate filing differed by sex, age, race/ethnicity, Minnesota region, living setting, location of death, hospitalization history, autopsy, variant era, and underlying conditions (Table 2). However, median time to death certificate filing did not exceed 7.5 days for any subgroup except decedents who underwent an autopsy (22 days) and decedents <18 years of age (40 days). Among decedents who did not undergo an autopsy, median time from DOD to death certificate filing was similar for those who were younger (<50 years, 4 days; <18 years, 2 days) and those who were >50 years of age (3 days). Median time from DOD to death certificate filing was longer for decedents who resided in an other setting (e.g., homeless shelter) (7.5 days) than for private or long-term care residents (3 days) (Table 2). Median time from DOD to death certificate filing was also longer for Asian/Pacific Islander and Black/African American decedents (6 days) than for White non-Hispanic decedents (3 days).

**Table 2:** Median days from date of death to filing of death certificate, by demographic and disease history characteristics, for confirmed COVID-19 deaths detected by using the Minnesota Department of Health COVID-19 mortality case definition, March 19, 2020–December 31, 2022*

**Patient characteristic**	No.	Days from date of death to death certificate filing, median (IQR)	p value
**Sex**			0.0002†
**M**	7,336	3 (2–6)	
**F**	6,255	3 (2–5)	
**Age, y**			<0.0001†
**0–17**	14	40 (4–95)	
**18–49**	529	6 (3–20)	
**50–59**	903	4 (2–7)	
**60–69**	1,883	4 (2–6)	
**70–79**	3,098	3 (2–5)	
**>80**	7,164	3 (1–5)	
**Race/ethnicity**			<0.0001†
**American Indian/Alaska Native**	232	5 (3–7)	
**Asian/Pacific Islander**	512	6 (3–14)	
**Black/African American**	668	6 (3–13.5)	
**Hispanic**	341	4 (2–7)	
**Multiracial**	60	4 (2–8)	
**Other or Unknown**	21	4 (3–5)	
**White, non-Hispanic**	11,757	3 (2–5)	
**Minnesota region**			<0.0001†
**Greater Minnesota**	6,501	3 (1,–4)	
**Minneapolis and Saint Paul metropolitan area**	7,090	4 (2–6)	
**Living setting**			<0.0001†
**Private residence**	6,880	3 (2–6)	
**Long-term care**	6,645	3 (1–5)	
**Other‡**	66	7.5 (3–19)	
**Location of death**			<0.0001†
**Hospital inpatient**	6,748	3 (2–6)	
**Congregate living**	5,245	3 (1–5)	
**Other§**	1,598	4 (2–10)	
**Hospitalization history**			<0.0001†
**Hospitalized**	8,657	3 (2–5)	
**No/unknown**	4,934	3 (1–5)	
**Autopsy status**			<0.0001†
**Yes**	343	22 (5–45)	
**No/unknown**	13,248	3 (2–5)	
**Variant era**			<0.0001†
**Pre-alpha**	6,697	3 (2–5)	
**Alpha**	778	3 (2–5)	
**Delta**	2,799	3 (2–6)	
**Omicron BA.1**	1,754	3 (2–6)	
**Omicron BA.2**	314	3 (2–5)	
**Omicron BA.4/5**	1,249	3 (2–5)	
**Underlying conditions**			0.0038†
**Yes**	12,921	3 (2–5)	
**No**	188	4 (2–8)	
**Unknown**	482	3 (2–6)	
**Total**	13,591	3 (2–5)	

*Values are no. (%) except as indicated. IQR, interquartile range.†Statistically significant at p = 0.05. p values are for median 1-way analysis. ‡Other includes sheltered and unsheltered homeless, jail/prison–dormitories, and other settings. §Other includes decedents who died at home, in the emergency department, and in other settings, such as at another private residence.

### Death Certificate Agreement

Overall, death certificates accurately captured 96% (13,108) of Minnesota confirmed COVID-19 deaths (Table 3). Death certificates for 483 (4%) COVID-19 deaths confirmed by the Minnesota mortality definition did not include COVID-19 keywords, and an additional 413 (3%) death certificates listed COVID-19 as a cause of death but were classified as COVID-19 ruled-out deaths by the Minnesota mortality definition.

**Table 3:** COVID-19 inclusion on the death certificate and the MDH case definition of COVID-19 mortality, March 19, 2020–December 31, 2022

**COVID-19 inclusion status**	Confirmed COVID-19 death using MDH COVID-19 mortality case definition	Non–COVID-19 death	Total
**“COVID-19” on death certificate (death certificate comparison method)**	13,108	413	13,521
**“COVID-19” not on death certificate**	483	0	483
**Total**	13,591	413	14,004

*MDH, Minnesota Department of Health.

Agreement between the Minnesota definition of COVID-19 mortality and inclusion of COVID-19 on the death certificate varied by demographics and disease history but was >90% for all groups analyzed except pediatric decedents (Appendix Table 1). Rate of agreement was highest for decedents who underwent an autopsy (98%), decedents with no underlying conditions (97%), and decedents who were identified as Asian/Pacific Islander (97%) (Appendix Table 1). Rate of agreement was lowest for pediatric decedents (<18 years of age) (74%), followed by decedents who died in congregate living (90%) and those with no known hospitalization history (90%). The median time from onset or positive specimen date to death was shorter for decedents with death certificate agreement (14.0 days, IQR 8.0–26.0 days) than for decedents with disagreement (19.0 days, IQR 6.0– 75.5 days).

## Discussion

We found that death certificates are accurate and timely sources of COVID-19 mortality surveillance data in Minnesota. However, agreement between death certificates and the Minnesota definition of COVID-19 mortality, as well as timeliness of death certificate filing, varied by certain demographics. In addition, access to provisional death certificates is a consistently expeditious method for obtaining reports of potential COVID-19–associated deaths.

In our analysis, median days between DOD and death certificate filing differed by multiple demographic and disease history variables. However, median time was within 8 days for most groups except those who underwent an autopsy (22 days) and pediatric decedents (40 days), enabling timely access to mortality data for public health insight. In addition, autopsies are rare, and delay is expected when they are performed. Median time from DOD to death certificate filing was longer for younger decedents (<50 years of age, particularly <18 years of age) than for those in older age groups, although the median time to death certificate filing for those who did not undergo autopsy was similar across age groups (<50 years, 4 days; <18 years, 2 days; ≥50 years, 3 days). Median time to death certificate filing was longer for those in Black, Indigenous, or People of Color (BIPOC) groups, potentially reflecting inequities in healthcare access or increased rates of autopsy (*19*–*22*).

Decedents with agreement between COVID-19 mortality definition and the death certificate were more likely to have a known hospitalization history or completed autopsy. Hospitalization and autopsy provide valuable information and context for both the health department and the certifier, probably contributing to greater agreement. That finding is consistent with a prior study in Olmsted County, Minnesota, that found higher rates of death certificate accuracy for coronary artery disease and an autopsy rate that was twice the national average (*23*).

Disagreement between the death certificate and the Minnesota mortality definition was more common among decedents <18 years of age, decedents who were White non-Hispanic, decedents who were long-term care residents, and decedents who died outside a hospital. The MDH UNEX/MED-X program investigates unexplained deaths of possible infectious etiology in addition to conducting population-based surveillance for deaths that may be associated with infectious disease(s) and are reported to Minnesota medical examiners (*15*,*16*). The program performs postmortem testing for an assortment of infectious diseases on a wide range of decedents, including medical examiner–investigated deaths that may have resulted from nonnatural causes. UNEX/MED-X identifies many young decedents, as well as many persons with incidental COVID-19 infection or nonnatural alternative causes of death (e.g., drug overdose), who may not have otherwise been identified by routine surveillance systems. Inclusion of COVID-19 as a contributing cause of death on the death certificate for young decedents with a nonnatural alternative cause of death probably explains the higher rate of disagreement among pediatric decedents. In contrast, BIPOC persons may be less likely to have a laboratory confirmed SARS-CoV-2 test result or to access healthcare for COVID-19 illnesses (*20*,*22*,*24*,*25*) and may therefore be more likely to be underreported in case surveillance, resulting in higher rates of death certificate agreement. BIPOC status has been associated with hospitalization risk (*26*–*31*), and non–English-speaking status and Black race have been associated with lower rates of SARS-CoV-2 testing (*32*,*33*).

Long-term care decedents were more likely to be considered both a confirmed COVID-19 death without death certificate and a ruled-out death when compared with private residents, resulting in a lower rate of agreement (Appendix Table 2). Those findings may result from multiple factors, including detection of mild or asymptomatic SARS-CoV-2 during facilitywide testing and complicated medical histories. Detection of mild or asymptomatic SARS-CoV-2 may increase the likelihood of COVID-19 being indicated on the death certificate in addition to an alternative cause of death. In addition, underlying health conditions (e.g., chronic obstructive pulmonary disease) are more prevalent in older and institutionalized populations (*34*), and such conditions can complicate cause-of-death determination. Residents may also be less likely to communicate subjective symptoms associated with COVID-19 infection or may exhibit signs not always associated with infection (e.g., falling more frequently). In addition, previous literature has found that death certificates overestimate death caused by coronary artery disease in certain demographic groups (*35*), particularly out-of-hospital deaths, which are common among long-term care residents (*36*). Other reports have found underestimates of Legionnaires’ disease by death certificates and misattribution of death to underlying conditions or other illnesses because of nonspecific manifestations (*37*). It is reasonable to assume a similar phenomenon may occur among COVID-19 case-patients with complex medical histories, particularly if symptoms could be confused for those of an existing chronic condition (e.g., shortness of breath) or are unobservable (e.g., loss of sense of taste).

Overall, death certificates were generally in high agreement with case-based investigation using the Minnesota definition of COVID-19 mortality. Undercounting (missing confirmed COVID-19 deaths) was observed more often than overcounting (i.e., erroneously identifying a decedent as a COVID-19 death). Those results are consistent with the existing literature (*3*–*7*). In our analysis, agreement ranged from 74% among pediatric decedents to 98% among those who underwent an autopsy; agreement was >90% for all groups except pediatric decedents. The small sample size for pediatric deaths complicates efforts to understand differences in characteristics. As more data become available, mortality surveillance systems should adapt as needed to better serve and identify special populations. Adaptations may include additional investigations into potential COVID-19 pediatric deaths (e.g., medical record review) to describe those deaths as accurately as possible.

Death certificates can be combined with case surveillance data (e.g., SARS-CoV-2 testing data) to form a robust COVID-19 mortality surveillance system. Misclassification of COVID-19 deaths is inevitable. Our analysis suggests that misclassification may skew toward underreporting and that some groups are more likely than others to be misclassified. Factors to consider when evaluating misclassification or cases missed by a surveillance system are racial and ethnic disparities in healthcare access, healthcare-seeking behavior, and infection and severe illness risk. For example, pediatric decedents may be overcounted because of intense scrutiny and comprehensive testing, and BIPOC persons may be undercounted because of lack of access to testing. Reliance on death certificates as a primary source of COVID-19–associated death reporting may serve as an accurate surveillance system, especially with limited public health resources. However, when available, public health entities should consider investing resources in more investigations for populations who may be more likely to be misclassified by death certificates, such as pediatric decedents and long-term care residents.

Among the limitations of our analysis, high reliance on the death certificates in the MDH COVID-19 death identification and determination process may skew results toward greater congruence between the death certificate and COVID-19 mortality definition. Although death certificates were an integral part of the Minnesota definition of COVID-19 mortality, we conducted further investigation for specified COVID-19 language, for deaths that occurred >1 year after a positive test result and for deaths that occurred within 30 days of a positive test result and did not have COVID-19 indicated on the death certificate. Therefore, our analysis compares a resource-intensive approach, including review of all available sources, with reliance on death certificate reporting alone. In addition, our analysis identified populations for which an intensive investigation was less likely to agree with the death certificate, which could have implications for future mortality surveillance and analysis.

The Minnesota definition of COVID-19 mortality underwent several revisions during the study period; specifically, a prior definition categorized deaths as probable if the decedent had undergone only antigen testing for SARS-CoV-2. However, probable and confirmed deaths were both still considered COVID-19 deaths and therefore did not affect the overall mortality reporting used for our analysis. In addition, resources to investigate deaths fluctuated throughout the pandemic, so it is possible that not all deaths underwent the same level of investigation. Because intensive investigations were used to rule out and rule in COVID-19 deaths, we expect that the net effect of resource differences over time would minimally affect our findings.

The Minnesota case definition was in place for several years from the beginning of the pandemic, resulting in consistent detailed data collection beyond the initial phase of intensive COVID-19 contact tracing and case investigations. Confirmed Minnesota COVID-19 deaths in our analysis are laboratory-confirmed COVID-19 cases. Documented inequities in access to COVID-19 testing (*32*,*33*,*38*) may have biased mortality and case surveillance. Access to (and use of) at-home tests, which are not reported to MDH or counted as laboratory-confirmed cases, has increased, particularly during the latter stages of the pandemic, and are probably used at different rates by different populations. The population of our analysis constitutes mostly White non-Hispanic persons in an upper Midwestern state with robust public health resources, including the UNEX/MED-X program, and thus, results may not be generalizable to other states. Variables assessed in our analysis may be correlated (e.g., long-term care residency and race/ethnicity), potentially affecting analysis outcomes and generalizability to states with different demographics. Future research may use regression models to explore the relationship between demographic and disease history variables.

Trained public health professionals in Minnesota evaluated death certificates for any mention of COVID-19 rather than searching for specific ICD codes or underlying causes of death, which enabled more timely death reporting and inclusion of language from the entire death certificate. That approach also avoided errors associated with incorrect identification of underlying cause of death on the death certificate, which previous research suggests may be common (*39*,*40*). Last, our analysis adds to the body of COVID-19 knowledge by evaluating a COVID-19 mortality surveillance approach for accuracy and timeliness, providing context for future mortality surveillance development and evaluation of emerging diseases in Minnesota and beyond.

In conclusion, we analyzed a large sample of possible COVID-19 deaths that were reviewed by using a standard case definition throughout several years of the COVID-19 pandemic. Comparisons of the Minnesota definition of COVID-19 mortality and inclusion of COVID-19 on the death certificate indicated that death certificates are an efficient and timely source of COVID-19 mortality data when paired with SARS-CoV-2 testing data and should be an integral part of COVID-19 mortality surveillance. Supplemental investigations may be warranted for key groups, such as pediatric decedents, as resources allow. Mortality surveillance is a vital aspect of disease surveillance, particularly during emergence of a new disease, and surveillance evaluations are needed to improve existing systems and prepare for the next public health challenge.

Appendix Additional information for study of COVID-19 death determination methods, Minnesota, USA, 2020–2022.
