# Supplementary material for: COVID-19 Death Determination Methods, Minnesota, USA, 2020–2022
Source: Emerg Infect Dis. 2024 Jul;30(7):1352–60. doi: 10.3201/eid3007.231522 (PMC11210668; doi:10.3201/eid3007.231522)
Supplement: Appendix — Additional information for study of COVID-19 death determination methods, Minnesota, USA, 2020–2022. [file 23-1522-Techapp-s1.pdf]

Article DOI: https://doi.org/10.3201/eid3007.231522

EID cannot ensure accessibility for supplementary materials supplied by authors. Readers who have difficulty accessing supplementary content should contact the authors for assistance.

COVID-19 Death Determination Methods, Minnesota, USA, 2020–2022

Appendix

COVID-19 Death Case Definitions as of 1/7/22 – 12/31/22

COVID-19 Death Case Definition of Confirmed Cases*

1. Decedent has a positive laboratory SARS-CoV-2 RNA PCR or a positive antigen test, AND
2. Meets at least ONE of the following criteria:
   a. Death certificate mentions COVID-19 (or equivalent term) in Part 1 or Part 2 of the death certificate[†].
   b. Clinical history or autopsy findings and epidemiologic evidence that death is related to SARS-CoV-2 infection, i.e.
      i. Signs or symptoms consistent with COVID-19 OR autopsy findings consistent with acute respiratory distress syndrome OR pneumonia without an alternative cause and decedent did not have a complete recovery back to baseline state of health

OR

      ii. Death occurred ≤ 30 days from specimen collection date of positive SARS-CoV-2 test, AND decedent did not have a fully explanatory alternative cause of death that was causally unrelated to SARS-CoV-2 infection (e.g., accident, homicide, drowning, etc.)

COVID-19 Death Case Definition of Death Certificate Only Cases

1. Decedent does NOT have a positive laboratory confirmed SARS-CoV-2 RNA PCR OR a positive antigen test‡, AND
2. Death certificate mentions COVID-19 (or equivalent term) in Part 1 or Part 2 of the death certificate†.

NOTE: Death investigations involve receiving and reviewing information from multiple sources, including but not limited to death certificates, medical records, case report forms, laboratory reports, and autopsy reports before a case classification can be made. Deaths are considered current and not part of any backlog if it is < 6 months between the date of death and the date the death is reported out.

*A prior case definitions distinguished between probable and confirmed COVID-19 deaths based on whether the decedent had a laboratory-confirmed PCR (confirmed) or antigen (probable) test. However, both confirmed and probable deaths were considered COVID-19 deaths for statewide mortality tracking and in the analyses performed in this study.

†For death certificates that list COVID-19 or equivalent in Part 1 or 2 of the death certificate and the death has been >30 days from positive test and/or the decedent returned to baseline state of health, MDH Infectious Disease Epidemiology, Prevention, and Control staff may notify the MDH Office of Vital Records staff and request that the medical certifier review the death certificate for accuracy.

‡For death certificates that list COVID-19 or equivalent in Part 1 or Part 2 of the death certificate and the death occurred ≤7 days from a negative test, MDH Infectious Disease Epidemiology, Prevention, and Control staff may notify the MDH Office of Vital Records staff and request that the medical certifier review the death certificate for accuracy.

Appendix Table 1. Death certificate and the Minnesota Department of Health COVID-19 mortality case definition agreement by demographic and disease history characteristics, March 19, 2020–December 31, 2022*

Characteristic	Total, no. (%)	Mortality definition and death certificate agreement, no. (%)	Mortality definition and death certificate disagreement, no. (%)	p-value
Sex				0.0002†
M	7,518 (53.7)	7,091 (94.3)	427 (5.7)	
F	6,486 (46.3)	6,017 (92.8)	469 (7.2)	
Age, y				<0.0001†
0 – 17 y old				0.0058†‡
Yes	19 (0.1)	14 (73.7)	5 (26.3)	
No	13,985 (99.9)	13,094 (93.6)	891 (6.4)	
18 – 49 y old				0.12
Yes	561 (4.0)	516 (92.0)	45 (8.0)	
No	13,443 (96.0)	12,592 (93.7)	851 (6.3)	
50 – 59 y old				0.0004†
Yes	920 (6.6)	885 (96.2)	35 (3.8)	
No	13,084 (93.4)	12,223 (93.4)	861 (6.6)	
60 – 69 y old				<0.0001†
Yes	1,921 (13.7)	1,841 (95.8)	80 (4.2)	
No	12,083 (86.3)	11,267 (93.3)	816 (6.8)	
70 – 79 y old				0.0068†
Yes	3,176 (22.7)	3,005 (94.6)	171 (5.4)	
No	10,828 (77.3)	10,103 (93.3)	725 (6.7)	
80+ years old				<0.0001†
Yes	7,407 (52.9)	6,847 (92.4)	560 (7.6)	
No	6,597 (47.1)	6,261 (94.9)	336 (5.1)	
Race/ethnicity				0.0060†
American Indian/Alaska Native				0.70
Yes	241 (1.7)	227 (94.2)	14 (5.8)	
No	13,763 (98.3)	12,881 (93.6)	882 (6.4)	
Asian/Pacific Islander				<0.0001†
Yes	517 (3.7)	503 (97.3)	14 (2.7)	
No	13,487 (96.3)	12,605 (93.5)	882 (6.5)	
Black/African American				0.64
Yes	686 (4.9)	645 (94.0)	41 (6.0)	
No	13,318 (95.1)	12,463 (93.6)	855 (6.4)	
Hispanic				0.20
Yes	353 (2.5)	336 (95.2)	17 (4.8)	
No	13,651 (97.5)	12,772 (93.6)	879 (6.4)	
Multiracial				1.00‡
Yes	62 (0.4)	58 (93.6)	4 (6.5)	
No	13,942 (99.6)	13,050 (93.6)	892 (6.4)	
Other/unknown				1.00‡
Yes	22 (0.2)	21 (95.5)	1 (4.6)	
No	13,982 (99.8)	13,087 (93.6)	895 (6.4)	
White, non-Hispanic				0.0021†
Yes	12,123 (86.6)	11,318 (93.4)	805 (6.6)	
No	1,881 (13.4)	1,790 (95.2)	91 (4.8)	
Region				0.26
Greater Minnesota	6,665 (47.6)	6,255 (93.9)	410 (6.2)	
Minneapolis and Saint Paul metropolitan area	7,339 (52.4)	6,853 (93.4)	486 (6.6)	
Living setting				<0.0001†
Private residence				<0.0001†
Yes	7,018 (50.1)	6,738 (96.0)	280 (4.0)	
No	6,986 (49.9)	6,370 (91.2)	616 (8.8)	
Long-term care				<0.0001†
Yes	6,916 (49.4)	6,306 (91.2)	610 (8.8)	
No	7,088 (50.6)	6,802 (96.0)	286 (4.0)	
Other§				0.46‡
Yes	70 (0.50)	64 (91.4)	6 (8.6)	
No	13,934 (99.5)	13,044 (93.6)	890 (6.4)	
Location of death				<0.0001†
Hospital inpatient				<0.0001†
Yes	6,827 (48.8)	6,616 (96.9)	211 (3.1)	
No	7,177 (51.3)	6,492 (90.5)	685 (9.5)	
Congregate living				<0.0001†
Yes	5,497 (39.3)	4,958 (90.2)	539 (9.8)	
No	8,507 (60.8)	8,150 (95.8)	357 (4.2)	

Characteristic	Total, no. (%)	Mortality definition and death certificate agreement, no. (%)	Mortality definition and death certificate disagreement, no. (%)	p-value
Other¶				<0.0001†
Yes	1,680 (12.0)	1,534 (91.3)	146 (8.7)	
No	12,324 (88.0)	11,574 (93.9)	750 (6.1)	
Hospitalization history				<0.0001†
Hospitalized	8,860 (63.3)	8,465 (95.5)	395 (4.5)	
No/unknown	5,144 (36.7)	4,643 (90.3)	501 (9.7)	
Autopsy status				0.0004†
Yes	343 (2.5)	335 (97.7)	8 (2.3)	
No	13,661 (97.6)	12,773 (93.5)	888 (6.5)	
Underlying conditions status				0.040†
Underlying conditions present				0.63
Yes	13,300 (95.0)	12,446 (93.6)	854 (6.4)	
No	704 (5.0)	662 (94.0)	42 (6.0)	
No underlying conditions present				0.015†
Yes	192 (1.4)	187 (97.4)	5 (2.6)	
No	13,812 (98.6)	12,921 (93.6)	891 (6.5)	
Unknown if underlying conditions present				0.44
Yes	512 (3.7)	475 (92.8)	37 (7.2)	
No	13,492 (96.3)	12,633 (93.6)	859 (6.4)	
Median specimen date to death (days, IQR)#	15.0 (8.0–27.0)	14.0 (8.0–26.0)	19.0 (6.0–75.5)	0.0009†
Total	14,004	13,108 (93.6)	896 (6.4)	

*Values are no. (%) unless otherwise indicated. IQR, interquartile range.
†Statistically significant at p-value = 0.05. p-values are for likelihood ratio chi-square or median one-way analysis unless otherwise specified.
‡p-value is from Fisher exact test.
§Other includes sheltered and unsheltered homeless, jail/prison, dormitories, and other settings.
¶Other includes decedents who died at home, in the emergency department, and in other settings, such as at another private residence.
#All negative values (specimen collections after death) were replaced with zero.

Appendix Table 2. Death certificate and the Minnesota Department of Health COVID-19 mortality case definition agreement with directionality for age, race/ethnicity, living setting, location of death, and hospitalization history, March 19, 2020 – December 31, 2022

Characteristic	Total	Death certificate confirmed COVID-19 death, no. (%)	Non-death certificate confirmed COVID-19 death, no. (%)	Ruled-out death, no. (%)	p-value
Age					<0.0001
0 – 17	19 (0.14)	14 (73.7)	0 (0)	5 (26.3)	
18 – 49	561 (4.0)	516 (92.0)	13 (2.3)	32 (5.7)	
50 – 59	920 (6.6)	885 (96.2)	18 (2.0)	17 (1.9)	
60 – 69	1,921 (13.7)	1,841 (95.8)	42 (2.2)	38 (2.0)	
70 – 79	3,176 (22.7)	3,005 (94.6)	93 (2.9)	78 (2.5)	
80+	7,407 (52.9)	6,847 (92.4)	317 (4.3)	243 (3.3)	
Race/Ethnicity					0.0090
American Indian/Alaska Native	241 (1.7)	227 (94.2)	5 (2.1)	9 (3.7)	
Asian/Pacific Islander	517 (3.7)	503 (97.3)	9 (1.7)	5 (1.0)	
Black/African American	686 (4.9)	645 (94.0)	23 (3.4)	18 (2.6	
Hispanic	353 (2.5)	336 (95.2)	5 (1.4)	12 (3.4)	
Multiracial	62 (0.4)	58 (93.6)	2 (3.2)	2 (3.2)	
Other or Unknown	22 (0.2)	21 (95.5)	0 (0)	1 (4.6)	
White, non-Hispanic	12,123 (86.6)	11,318 (93.4)	439 (3.6)	366 (3.0)	
Living setting					<0.0001
Private residence	7,018 (50.1)	6,738 (96.0)	142 (2.0)	138 (2.0)	
Long-term care	6,916 (49.4)	6,306 (91.2)	339 (4.9)	271 (3.9)	
Other*	70 (0.5)	64 (91.4)	2 (2.9)	4 (5.7)	
Location of death					<0.0001
Hospital inpatient	6,827 (48.8)	6,616 (96.9)	132 (1.9)	79 (1.2)	
Congregate living	5,497 (39.3)	4,958 (90.2)	287 (5.2)	252 (4.6)	
Other†	1,680 (12.0)	1,534 (91.3)	64 (3.8)	82 (4.9)	

Characteristic	Total	Death certificate confirmed COVID-19 death, no. (%)	Non-death certificate confirmed COVID-19 death, no. (%)	Ruled-out death, no. (%)	p-value
Hospitalization history					<0.0001
Hospitalized	8,860 (63.3)	8,465 (95.5)	192 (2.2)	203 (2.3)	
No/unknown	5,144 (36.7)	4,643 (90.3)	291 (5.7)	210 (4.1)	
Total	14,004	13,108 (93.6)	483 (3.5)	413 (3.0)	

*Other includes sheltered and unsheltered homeless, jail/prison, dormitories, and other settings.
†Other includes decedents who died at home, in the emergency department, and in other settings, such as at another private residence.
p-values are for likelihood ratio chi-square.
